# Strengthening Rural Primary Healthcare for Climate Resilience in Zambia: An Equity‐Centered Approach

**DOI:** 10.1002/puh2.70390

**Published:** 2026-09-29

**Authors:** Newton Nyirenda, Hannah Muturi, Lweendo Muchaili, Matenge Mutalange, Collins Himabala, Donald Kaoma, Raymond Ernest, Caren Mbulo, Jackson Shawa, Linda Siachalinga

**Affiliations:** ^1^ Chikankata District Health Office Ministry of Health Chikankata Zambia; ^2^ University of the District of Columbia Washington District of Columbia USA; ^3^ Mulungushi University Livingstone Zambia; ^4^ WaterAid Zambia Lusaka Zambia; ^5^ Karolinska Institutet Stockholm Sweden; ^6^ Chilenje First Level Hospital Ministry of Health Lusaka Zambia; ^7^ University of Zambia Lusaka Zambia; ^8^ Griffith University Gold Coast Queensland Australia

**Keywords:** climate adaptation, climate resilience, health equity, infrastructure, primary healthcare, rural health systems, supply chain, universal health coverage, water, sanitation

## Abstract

Climate change presents an escalating threat to health systems worldwide, with rural populations in low‐income countries disproportionately affected. In Zambia, where primary healthcare (PHC) is the main access point for most communities, extreme weather events and environmental degradation increasingly disrupt service delivery and threaten health outcomes. However, limited evidence exists on how these climate risks intersect with systemic vulnerabilities in rural health systems. We explored the pathways through which climate change disrupts rural PHC delivery in Zambia and identified priority domains for adaptation and resilience‐building. We conducted a narrative synthesis of national data, peer‐reviewed literature, and regional case studies to assess climate‐related risks affecting rural health systems. The analysis focused on five critical domains: infrastructure damage, energy insecurity; water, sanitation, and hygiene (WASH) vulnerabilities; supply‐chain disruptions; and geographic accessibility. Findings were contextualized within Zambia's rural health landscape and aligned with the World Health Organization (WHO) framework on climate‐resilient health systems. Rural PHC services in Zambia face intersecting climate risks, including flood‐induced infrastructure damage, widespread energy and water insecurity, disrupted supply chains, and reduced access to care during extreme weather. These challenges are compounded by structural weaknesses, such as limited off‐grid infrastructure, seasonal transport barriers, and insufficient integration of climate resilience into national and district health planning. Vulnerable populations, especially women, children, and those in remote areas, experience the greatest barriers under climate stress. Strengthening climate resilience in Zambia's PHC system requires urgent, equity‐focused interventions across infrastructure, energy, WASH, and logistics. Mainstreaming climate adaptation into health policy and planning is essential to protect vulnerable populations and sustain health service delivery. Enhancing PHC resilience is not only critical for climate preparedness but also central to achieving universal health coverage and long‐term health system sustainability.

## Graphical abstract

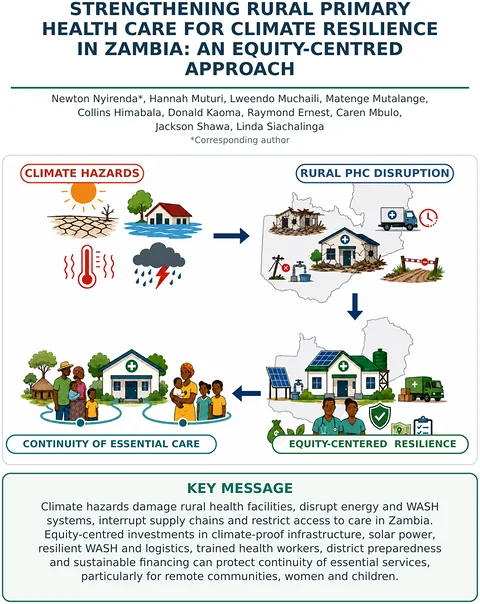

## 1 Introduction

Health systems in low‐ and middle‐income countries (LMICs) are increasingly challenged by the convergence of two powerful trends: the rising frequency and severity of climate‐related shocks (such as floods, droughts, heat waves, and storms) and the persistent struggles of delivering essential primary care in rural, under‐resourced settings [1, 2, 3]. In sub‐Saharan Africa (SSA), many health systems are reported to be under‐prepared for climate‐related risks in areas of infrastructure resilience, workforce capacity, surveillance, and service continuity [4].

Within this broad field, there remains a pronounced gap in attention paid to rural primary healthcare (PHC) facilities as frontline assets. Much of the current literature focuses on national systems or urban hospitals, rather than the rural clinics that often deliver care to the most underserved populations [5, 6]. For instance, Lokotola et al. observed that although climate–health linkages are increasingly documented in Africa, “there is a lack of evidence on climate‐resilient PHC” in rural settings [7].

Evidence from empirical studies and regional assessments suggests that rural health infrastructure is exposed to multiple, often interlinked pathways of vulnerability: interruption of power, water, and sanitation services; damage to physical buildings caused by extreme weather; breakdowns in transport and logistics; disrupted cold chains; and reduced availability of skilled providers due to remoteness [8, 9]. For example, a facility‐level vulnerability assessment in a rural hospital in Chad found significant infrastructure risks, such as unreliable power supply and environmental hazards that undermined service delivery [6].

The broader significance of climate change for African health systems is increasingly clear: It is described as “the greatest health threat of the 21st century” in the African context, threatening to reverse gains in maternal and child health, infectious disease control, and progress toward universal health coverage (UHC) and the Sustainable Development Goals (SDGs) [10, 11, 12, 13]. Rural communities are particularly vulnerable because poverty, limited educational opportunities, weak transport networks, and health‐workforce shortages compound their exposure to climate‐related risks [13, 14, 15].

Energy access is a critical enabler of health infrastructure resilience and quality of care in rural settings [16, 17, 18, 19]. Evidence from SSA shows that inadequate electricity supply in rural health facilities directly undermines essential service delivery. A study of rural health facilities in Kenya, Ghana, and Rwanda found that poor electricity supply is associated with compromised service delivery, especially for neonatal care and vaccine cold‐chain maintenance [9, 20]. Moreover, a recent report on powering healthcare in the Global South highlighted the “double threat” posed by climate change: healthcare facilities face both increased demand (from climate‐related health burdens) and increased disruption (to their energy supply and infrastructure) [21].

Policy reviews and secondary literature suggest that important gaps persist in climate adaptation planning for rural health systems. Many health system strengthening frameworks do not explicitly integrate infrastructure resilience, climate adaptation, or rural‐specific primary care delivery [11, 22, 23]. Reviews point out that governance, financing, cross‐sectoral coordination (health, environment, transport, and infrastructure), and infrastructure adaptation in the context of climate risk remain nascent domains in SSA [22, 23]. There is emerging recognition of conceptual frameworks for climate‐resilient health systems, such as by Perera et al., yet empirical work remains scant, especially for rural PHC infrastructure [23, 24].

Zambia enters this period of climate stress with substantial baseline infrastructure deficits. The 2018 Zambia Demographic and Health Survey reported that approximately 8% of rural households had electricity access [25]. Facility‐level evidence is similarly concerning: Analysis of a nationally representative Zambian health‐facility survey found that only 35% of rural health centers had a functional grid connection [26]. These deficits threaten lighting, diagnostics, communication, refrigeration, and emergency care when electricity systems or transport networks are disrupted. Published Zambian studies that directly quantify climate‐attributable interruptions in rural PHC remain scarce. Despite the evident need, very few studies have examined how rural health infrastructure (facility buildings, energy, water, and transport/logistics) can be systematically strengthened for climate resilience in Zambia or comparable low‐resource rural settings. This gap presents both a research opportunity and a policy imperative.

Therefore, this article aims to (1) analyze the pathways by which climate‐related risks disrupt rural health service delivery in Zambia's PHC settings; (2) identify critical infrastructure and system vulnerabilities; and (3) propose actionable, scalable policy interventions aligned with Zambia's health, environment, and development agendas.

## 2 Methods

### 2.1 Review Design and Analytical Framework

This study used a narrative review and policy‐synthesis design to examine how climate‐related hazards affect rural PHC service continuity in Zambia. The purpose was not to generate pooled effect estimates but to integrate empirical studies, national policy documents, and technical reports to identify recurring vulnerability pathways and feasible policy actions. The analysis was guided by the World Health Organization (WHO) operational framework for building climate‐resilient health systems, with particular attention to infrastructure, essential environmental services, energy, supply chains, workforce preparedness, governance, and equity [5].

### 2.2 Information Sources and Search Strategy

Peer‐reviewed literature was identified through PubMed/MEDLINE and Google Scholar. Grey literature and policy documents were located through targeted searches of the websites of the Zambia Ministry of Health (MoH), Ministry of Green Economy and Environment, Zambia Statistics Agency, WHO, WHO Regional Office for Africa, UNICEF, World Bank, United Nations Development Programme (UNDP), and other relevant multilateral organizations. The core search combined terms for hazards, health systems, and geography: (“climate change” OR flood* OR drought* OR heat wave* OR “extreme weather”) AND (“primary health care” OR “primary healthcare” OR “health facility*” OR clinic* OR “health system*”) AND (Zambia OR rural OR “sub‐Saharan Africa”). Supplementary searches combined these terms with electricity; solar energy; water, sanitation, and hygiene (WASH); supply chain; cold chain; transport; referral; access; maternal and child health; equity; resilience; and adaptation. Reference lists of included sources were also screened.

### 2.3 Eligibility, Selection, and Synthesis

Eligible sources were English‐language publications issued mainly from 2010 to 2025 that addressed at least one of the following: climate‐related disruption to PHC or health facilities; rural health infrastructure, energy, WASH, transport, referral, or supply continuity; climate‐resilient health‐system policy and planning; or equity implications for underserved populations. Foundational Zambian policy documents published before 2010 were retained when directly relevant. Zambia‐specific empirical evidence and official national documents were prioritized. Evidence from other SSA or comparable LMIC settings was included only where national evidence was limited and the context was plausibly transferable. Sources focused solely on disease burden without a health‐system or service‐delivery component, exclusively urban or tertiary settings without relevance to rural PHC, opinion pieces without supporting evidence, duplicates, and documents with insufficient methodological or policy detail were excluded. Potentially relevant titles, abstracts, executive summaries, and full texts were reviewed for relevance. Information was synthesized by setting, hazard, health‐system domain, service effect, equity implication, and proposed adaptation. Findings were grouped into five domains: infrastructure damage, energy insecurity, WASH vulnerability, supply‐chain disruption, and geographic or transport inaccessibility. Regional examples are presented as contextual evidence rather than direct estimates for Zambia. No formal risk‐of‐bias assessment or meta‐analysis was undertaken.

## 3 Climate‐Related Risks and Pathways of Disruption in Rural Health Service Delivery

Climate change presents mounting threats to rural health systems, particularly in LMICs like Zambia, where fragile infrastructure, workforce shortages, and geographic inaccessibility already compromise service delivery. Rural health services are uniquely vulnerable to climate‐related shocks that disrupt physical access, damage essential infrastructure, interrupt supply chains, and exacerbate water and energy insecurity. These risks are not only immediate in their impact but also cumulative, reinforcing long‐standing inequities in maternal and child health outcomes [1, 27, 28, 29].

Extreme weather events increasingly threaten rural health infrastructure across SSA [3, 6]. In rural Zambia, seasonal flooding and heavy rainfall can inundate travel routes, make unpaved roads impassable, and substantially delay or prevent access to health facilities [30]. An official Zambian ministerial statement on the 2021/2022 rainy season reported that 107 bridges and crossing points had been damaged or washed away and that 46 health posts had lost their roofs during floods, storms, and heavy rainfall [31]. This provides direct national evidence of infrastructure exposure, although the report does not quantify the duration of facility closure or the volume of services lost. Comparable facility‐level evidence from rural Chad demonstrates how climate‐related environmental hazards can compromise essential services, including maternity and neonatal care [6].

Energy insecurity is a major Zambia‐specific vulnerability. A nationally representative health‐facility survey reported that only 35% of rural health centers had a functional connection to the electricity grid [26]. Other national reports suggest that more than 70% of rural health facilities in Zambia have reliable electricity access, with a smaller fraction connected to renewable backup systems, such as solar [32]. Across SSA, many health facilities also lack reliable electricity, creating bottlenecks for lighting, diagnostics, communication, sterilization, emergency procedures, and cold‐chain continuity [19]. These service interruptions are compounded by widespread energy insecurity. Consistent power supply is critical for cold‐chain maintenance, diagnostics, emergency procedures, and nighttime deliveries. Several studies have highlighted the tangible health impacts of energy gaps, noting that outages during extreme weather directly impede maternal and newborn service provision [33, 34].

WASH systems are similarly compromised by climatic extremes. During droughts, rural clinics may experience dry boreholes and water rationing, whereas during floods, pit latrines often overflow, contaminating nearby water sources [35, 36]. UNICEF Zambia reports that 13% of health care facilities lack any water service [37]. Such deficits directly impact infection control, antenatal care quality, and resilience to outbreaks, such as cholera or typhoid. Inadequate water and sanitation undermine infection prevention, safe maternity care, environmental cleaning, and outbreak response. Globally, approximately one in four health facilities lacks basic water services, with the greatest deficits concentrated in low‐resource settings [38].

Supply chains, particularly for vaccines, oxytocin, and essential medicines, are frequently disrupted during extreme weather events. Impassable roads and washed‐out bridges delay replenishment cycles, leaving health workers without key supplies during peak demand [39, 40, 41]. Droughts may also increase the need for nutritional supplements and oral rehydration solutions, further straining limited inventories. Case studies in SSA have shown that climate‐linked supply disruptions significantly contribute to increased maternal and perinatal complications in rural zones [27, 42].

Perhaps most critically, climate change exacerbates geographic and transportation‐related barriers to care. A mixed‐methods study among women living in remote rural Zambia identified long distances, poor roads, transport costs, and limited vehicle availability as barriers to facility delivery [43]. During floods, routes become submerged or impassable, increasing home deliveries and delaying emergency obstetric referrals. The WHO Regional Office for Africa has emphasized that resilient transport networks must be central to national climate‐health adaptation strategies [44]. However, many national health plans have yet to incorporate road and bridge climate‐proofing into their rural health investments [45, 46].

Together, these climate‐sensitive vulnerabilities may undermine progress in expanding equitable rural health access. Without targeted adaptation strategies, such as elevating clinic foundations, solarizing health posts, reinforcing WASH systems, and investing in climate‐resilient transport infrastructure, rural health systems will remain on the frontline of climate‐induced disruption. Strengthening climate resilience in these domains is not merely an infrastructure challenge but a public health imperative to protect the most vulnerable communities from compounded harms. The major pathways through which climate change disrupts rural PHC delivery in Zambia are summarized in Table 1.

**TABLE 1:** Climate disruption pathways in rural health systems.

Disruption domain	Climate‐related risk	Service impact
Infrastructure damage	Floods, heavy rain, wind damage facility buildings	Clinic closure; suspension of maternal/emergency care
Energy insecurity	Heat waves increase energy demand; storms damage power sources	Cold‐chain breakdown; delivery complications in darkness
WASH vulnerabilities	Droughts/floods disrupt water availability and sanitation systems	Increased infection risk; poor hygiene and maternal care
Supply‐chain disruptions	Flooded/damaged roads delay delivery of medicines, vaccines	Stockouts of essential drugs and vaccines
Geographic/Transport access	Flooded or impassable terrain hinders access to clinics	Delayed care‐seeking; rise in home deliveries, worse outcomes

Abbreviation: WASH, water, sanitation, and hygiene.

## 4 Policy and Planning Gaps in Climate‐Resilient Health Infrastructure

Over the past decade, Zambia has developed a series of national strategies to address climate change and its sectoral impacts, including in health. The National Climate Change Response Strategy (NCCRS) launched in 2010 outlines goals to “climate‐proof infrastructure” and safeguard public health under changing environmental conditions [47]. Subsequent instruments, including the 2016 National Policy on Climate Change (NPCC) and the 2023 National Adaptation Plan (NAP), have reaffirmed the country's intent to integrate climate risk management across sectors [48, 49]. On the health side, the National Health Strategic Plan 2022–2026 prioritizes UHC and health systems strengthening, with an emerging emphasis on resilience [50]. Partnerships with global organizations have supported technical capacity‐building, and initiatives are underway to pilot climate‐resilient health facility models [51].

Despite this progress, policy analyses and implementation reports indicate several important planning and operational gaps that impede effective adaptation at the rural primary care level. First, although national frameworks mention infrastructure and health vulnerability, they provide little specific guidance on how to climate‐proof rural health posts and clinics. As observed in other low‐income countries, national plans often lack operational clarity on infrastructure adaptation and service continuity at the sub‐national level [52, 53].

Second, implementation of climate‐health strategies appears uneven in practice, particularly at district and facility levels. Although Zambia's NAP includes health sector actions, there is limited evidence of coordinated, budgeted investments in rural facility upgrading or adaptation [48, 53]. Reports from rural provinces continue to highlight recurring service disruptions due to floods, energy outages, and facility damage, suggesting that policy commitments have yet to translate into widespread implementation [49, 54].

Third, multisectoral coordination, a cornerstone of climate resilience, is often fragmented. Effective adaptation in rural settings requires collaboration across health, energy, transport, and infrastructure sectors. Yet, as with many countries in SSA, policy documents and implementation reports provide limited evidence of formalized institutional mechanisms to align health, transport, energy, and infrastructure adaptation efforts at district and facility levels in Zambia [55]. For instance, rural health infrastructure decisions are rarely synchronized with road planning or energy access strategies, creating systemic inefficiencies and missed opportunities for integrated resilience building [49].

Fourth, monitoring and evaluation systems for climate resilience in health infrastructure remain underdeveloped. National policies refer to tracking indicators but do not define climate‐resilient metrics at the facility level, such as percentage of rural clinics with backup power, flood‐resistant design, or climate vulnerability assessments completed. Without such indicators, it becomes difficult to assess progress, allocate resources, or respond proactively to emerging threats [48].

Finally, there is an absence of dedicated financing streams for climate‐resilient health infrastructure. The policy and planning documents reviewed did not identify a clearly defined and traceable financing stream dedicated specifically to climate‐resilient rural health‐facility adaptation. This omission is critical, as building resilience often requires up‐front investment in structural design, renewable energy systems, water security, and early warning integration. In the absence of targeted financing mechanisms, rural facilities remain vulnerable to predictable and preventable climate‐related service failures [47, 49, 50].

These policy and planning gaps may limit the health system's ability to provide uninterrupted care in rural Zambia during climate stress. They threaten to widen health inequities between rural and urban populations and undermine progress toward health‐related SDGs.

## 5 Policy Recommendations for Climate‐Resilient Rural Health Infrastructure

Building a climate‐resilient rural health system in Zambia requires a holistic and intersectoral approach grounded in both global best practices and local realities. Table 2 presents a framework of recommended interventions to improve climate resilience within Zambia's rural PHC system.

**TABLE 2:** Policy recommendations for climate‐resilient rural health infrastructure in Zambia.

Policy recommendation	Justification	Responsible actors	Expected outcomes
1. Develop a national rural health facility resilience strategy	Current national policies are too broad and lack rural‐specific guidance	Ministry of Health (MoH), Ministry of Green Economy and Environment	Clear targets and budget frameworks for rural health infrastructure resilience
2. Integrate climate risk indicators into health information systems	Tracking resilience indicators will support evidence‐based planning and accountability	MoH, Zambia Statistics Agency, DHIS2 teams	Real‐time tracking of facility vulnerabilities and service continuity risks
3. Electrify rural health facilities using solar microgrids	Unreliable grid access disrupts care delivery and cold chains in rural clinics	Rural Electrification Authority, MoH, Development Partners	Stable energy access improves care quality, delivery safety, and storage
4. Climate‐proof infrastructure in flood‐ and drought‐prone districts	Facilities in high‐risk regions require structural upgrades to maintain operations	Ministry of Infrastructure, MoH, Provincial Health Offices	Reduced service interruptions during floods/droughts in high‐risk areas
5. Create a cross‐sectoral rural health resilience task force	Lack of coordination between health, infrastructure, and environment sectors	Cabinet Office, MoH, Ministries of Infrastructure and Environment	Improved coordination and efficient use of climate adaptation resources
6. Establish a climate‐resilient infrastructure financing mechanism	Resilience investments require predictable, earmarked funds beyond donor projects	Ministry of Finance, Climate Funds, National Treasury	Dedicated financing pipeline for resilient infrastructure upgrades
7. Train health workers in climate adaptation preparedness	Health workers need skills to anticipate, respond to, and recover from climate shocks	Health Professions Council of Zambia, Training Institutions	Prepared and responsive health workforce at the frontline
8. Embed climate‐resilience in district health plans	Districts are operational units for health delivery but lack climate‐aligned mandates	Provincial and District Health Offices	Localized, actionable climate adaptation plans implemented at district level

Although national frameworks, such as Zambia's NAP and the NCCRS, have acknowledged health sector vulnerability, they often stop short of translating this recognition into operational strategies tailored to rural infrastructure needs [47, 49, 50]. To move from intention to impact, targeted investments and governance reforms must address infrastructure, energy, human resources, planning, and coordination.

One of the foundational steps is the development of a dedicated national strategy for rural health facility resilience. Unlike general policy documents, such a strategy would define infrastructure benchmarks, prioritize high‐risk provinces, and guide budget allocation for adaptation at the facility level. Comparable strategies in countries, like Madagascar, have demonstrated the importance of localized, climate‐aware health system planning that links vulnerability assessments to investment decisions [56, 57].

Accurate and timely data are essential for climate adaptation, yet many health information systems do not currently track facility‐level risks. Integrating climate resilience indicators into Zambia's existing District Health Information System (DHIS2), such as tracking days of power outage, seasonal facility access, or the percentage of clinics with solar backup, would allow routine monitoring and more responsive planning. The WHO and other international bodies have emphasized the importance of embedding environmental and infrastructure metrics into health information systems to support system strengthening and accountability [5, 58].

Among the most pressing needs is the expansion of reliable electricity access. Rural health posts in Zambia frequently suffer from erratic grid connections, undermining cold‐chain management, diagnostic services, and safe deliveries, particularly at night [59]. Evidence from Kenya, Rwanda, and Malawi shows that solar microgrids with battery storage are not only feasible but also cost‐effective and scalable in low‐resource settings [44, 60, 61]. Electrifying rural clinics through renewable sources would simultaneously strengthen service continuity and contribute to Zambia's broader energy sustainability goals.

Infrastructure adaptation must also account for geographic risk. Facilities in flood‐prone or drought‐affected regions, such as Luapula and Southern provinces, face recurrent damage and service interruptions. Simple but targeted upgrades, such as elevating buildings, waterproofing, and reinforcing structures, may dramatically improve facility operability during extreme weather [6, 57]. In Madagascar, a health system strengthening initiative that included such upgrades led to uninterrupted care delivery following a cyclone event [57]. This underscores the value of localized design and risk‐informed investment.

Coordination across ministries is equally essential. Resilient health infrastructure cannot be achieved by the MoH alone; it requires collaboration with ministries responsible for energy, transport, environment, and finance. Establishing a cross‐sectoral rural health resilience task force could provide a structured mechanism for aligning priorities, streamlining budgets, and reducing redundancies. WHO‐AFRO's 2024 regional framework identifies such coordination platforms as a key enabler of effective adaptation planning [5, 44].

However, planning without financing yields limited progress. The documents reviewed provide limited evidence of a dedicated and publicly traceable financing mechanism for climate‐resilient rural health infrastructure. Although external partners, such as the Global Fund and Green Climate Fund, may offer support, domestic allocation is critical to sustain long‐term resilience. The creation of an infrastructure adaptation fund, specifically for rural clinics, could enable scalable investment in energy, structural upgrades, water access, and logistics [62].

Human resources are another pillar of resilience. Rural health workers face increasing demands during climate‐related disruptions, including transport failures, stockouts, and higher climate‐sensitive disease burdens. Published Zambia‐specific evidence on health‐worker competencies and training in climate adaptation is limited, whereas regional studies identify important preparedness and knowledge gaps [63, 64, 65]. Incorporating climate modules into both pre‐service and in‐service training would equip providers to anticipate and manage service disruptions [66, 67, 68]. Pilot programs in Ethiopia and Ghana suggest that even brief training can improve staff confidence, response speed, and community communication during extreme events [69, 70].

Finally, climate resilience must be integrated into routine district health planning. District health offices are responsible for implementing services across vast rural areas. However, the reviewed national documents provide limited operational detail on how climate‐risk assessments, facility adaptation measures, and service‐continuity arrangements should be incorporated into annual district plans and budgets [22, 23]. Mandating climate risk assessments and infrastructure resilience strategies within district health operational plans would ensure that adaptation is not an isolated effort but part of the everyday governance of health delivery [57].

## 6 Discussion

This review indicates that climate resilience in rural Zambia is fundamentally a health‐system and equity issue rather than an infrastructure problem alone. Low baseline access to household and facility electricity, established transport barriers in remote communities, and limited operational detail in national policies create conditions in which floods, droughts, heat, and storms may cause disproportionate service disruption. The strongest available evidence supports immediate action on reliable energy, water security, accessible referral routes, resilient logistics, and facility‐level preparedness. However, the available evidence does not support precise national estimates of climate‐attributable clinic closures, stockouts, or water and sanitation failures. Regional studies therefore identify plausible mechanisms and potential interventions but should not be interpreted as direct evidence of effects in Zambia. Implementation is also likely to be affected by limited fiscal space, competing health priorities, shortages of technical personnel, maintenance weaknesses, and fragmented institutional responsibilities. A phased approach should therefore prioritize high‐risk facilities and interventions that protect essential services while generating local implementation and cost data.

### 6.1 Strengths and Limitations

A strength of this review is its integration of Zambia‐specific empirical evidence, national policy documents, and regional literature within an explicit rural equity framework. However, several limitations should be considered. First, a narrative review does not provide the exhaustive searching, duplicate screening, formal risk‐of‐bias appraisal, or pooled estimates expected of a systematic review; relevant studies may therefore have been missed, and source selection involved author judgment. Second, Zambia‐specific facility‐level evidence was sparse and uneven across provinces, requiring cautious use of regional and grey‐literature evidence. Third, the included sources were heterogeneous in design, setting, outcome definitions, and publication quality. Fourth, many national datasets were cross‐sectional or predated recent climate shocks and cannot establish that climate change caused a particular service interruption. Finally, this review assessed policy content and plausible implementation options rather than programmed effectiveness, feasibility, or cost‐effectiveness. The recommendations should therefore be tested through facility‐level vulnerability assessments, implementation research, economic evaluation, and routine monitoring.

## 7 Conclusion

Climate‐related hazards pose an increasing threat to the continuity of rural PHC services in Zambia and comparable low‐resource settings. The available evidence identifies documented and plausible disruption pathways involving infrastructure damage, energy and water insecurity, supply‐chain disruption, and reduced geographic access, with disproportionate consequences for underserved populations. These vulnerabilities are not evenly distributed but disproportionately affect underserved and hard‐to‐reach populations, deepening existing inequities in health outcomes.

To build resilient PHC systems, Zambia's health sector must prioritize climate adaptation as a core component of health planning. This includes integrating risk assessments into infrastructure development, scaling solar and off‐grid energy solutions, strengthening water and sanitation systems, and climate‐proofing supply and transport networks. Strategic investments in early warning systems, real‐time logistics coordination, and inclusive governance mechanisms can further buffer health systems against climate shocks.

Importantly, national policies must center equity by ensuring that climate‐resilience efforts prioritize the most vulnerable communities, those already experiencing compounded barriers to care. As the frequency and intensity of extreme weather events increase, the cost of inaction will continue to rise, both in human and economic terms. Strengthening climate‐resilient PHC is not only a public health imperative but also a foundational step toward achieving UHC and the SDGs in an era of climate uncertainty.

This article contributes to the climate‐health literature by highlighting rural PHC infrastructure as a critical determinant of climate resilience in low‐resource settings. Unlike prior work focused mainly on national systems or tertiary facilities, this synthesis emphasizes how climate‐related disruptions worsen existing rural inequities in energy access, transport, WASH systems, and continuity of care in Zambia.

Important research gaps remain, particularly regarding the effectiveness and feasibility of adaptation strategies such as solar electrification, flood‐resistant infrastructure, and climate‐informed district health planning. Further implementation research is needed to understand how rural health systems can operationalize climate resilience under real‐world resource constraints.

## Author Contributions

**Newton Nyirenda**: conceptualization, writing – original draft. **Hannah Muturi**: writing – review and editing. **Lweendo Muchaili**: writing – review and editing. **Matenge Mutalange**: writing – review and editing. **Collins Himabala**: writing – review and editing. **Donald Kaoma**: writing – review and editing. **Raymond Ernest**: validation, writing – review and editing. **Caren Mbulo**: writing – original draft, writing – review and editing. **Jackson Shawa**: writing – original draft, writing – review and editing. **Linda Siachalinga**: writing – original draft, writing – review and editing.

## Funding

The authors have nothing to report.

## Ethics Statement

The authors have nothing to report.

## Conflicts of Interest

The authors declare no conflicts of interest.

## Data Availability

No new datasets were generated or analyzed for this narrative review. All evidence is available from the cited publications and policy documents.
